# “A day in the life” – telemedicine in family medicine and its relationship with practicing physicians’ satisfaction: a cross-sectional study

**DOI:** 10.1186/s13584-024-00624-w

**Published:** 2024-07-29

**Authors:** Galia Zacay, Limor Adler, Yochai Schonmann, Joseph Azuri, Ilan Yehoshua, Shlomo Vinker, Anthony D Heymann, Shani Afek, Avivit Golan Cohen, Ilan Green, Robert Hoffman, Michal Shani

**Affiliations:** 1 Department of Family Medicine, Faculty of Medicine & Health Sciences, Tel Aviv University, Tel Aviv, Israel; 2 Department of Family Medicine, Meuhedet Healthcare Maintenance Organization, Tel Aviv, Israel; 3 Department of Family Medicine, Maccabi Healthcare Services, Tel Aviv, Israel; 4 Siaal Research Center for Family Medicine and Primary Care, Faculty of Health Sciences, Ben-Gurion University of the Negev, Beer-Sheva, Israel; 5 Department of Quality Measurements and Research, Clalit Health Services, Tel Aviv, Israel; 6 Medical Division, Leumit Healthcare Services, Headquarters, Tel Aviv, Israel; 7 Department of Family Medicine Sharon-Shomron District, Clalit Health Services, Kfar- Sava, Israel; 8 Department of Family Medicine Central District, Clalit Health Services, Rehovot, Israel

**Keywords:** Telemedicine, Remote visits, Primary care, Satisfaction, Burnout

## Abstract

**Background:**

Telemedicine has expanded rapidly in recent years, and many encounters that were conducted in person now take place remotely. This study aimed to assess primary care physicians’ (PCPs) attitudes towards the different modalities of patient care.

**Methods:**

This is a cross-sectional nationwide descriptive study conducted in Israel. We asked PCPs to document an entire workday and answer a short questionnaire after each visit. The questions addressed the type of visit (face-to-face, remote synchronous [telephone/video], or remote asynchronous [online requests]), the perceived quality of the visit, and the physicians’ feelings at the end of each visit. Before documenting their working day, we asked the participants to answer a questionnaire about their general attitudes toward different modalities of medical visits and how they affect their well-being and burnout.

**Results:**

Sixty physicians documented 2,025 visits, of which 39% took place in person, 36% stemmed from online patient requests, 18% were telephone meetings, < 1% were video meetings, and 6% consisted of other types of contact. Mixed effects logistic regressions were used to model the visits’ evaluation. The odds ratios (ORs) for perceived medical quality of visits focused on medical tasks were lower for non-face-to-face visits: OR = 0.39, 95% CI 0.25–0.59 for remote synchronous, and OR = 0.14, 95% CI 0.09–0.23 for remote asynchronous. The perceived medical quality of visits focused on administrative tasks was lower for remote asynchronous than for face-to-face visits (OR = 0.31, 95% CI 0.14–0.65). We found no association between medical quality and patients, physicians, or clinic characteristics. The inappropriateness of the visit modality was also associated with lower medical quality (OR = 0.13, 95% CI 0.09–0.18). We found a correlation between perception of medical quality and physicians’ feelings at the end of the visits, Spearman’s *r* = 0.82 (*p* < 0.001).

**Conclusions:**

A substantial portion of the visits was dedicated to administrative tasks and remote medicine. In comparison, physicians rated face-to-face visits’ quality higher than remote visits. Policymakers should intervene to minimize administrative work, reduce PCPs’ administrative workload, and direct patients to the optimal visit modality for their complaints. These steps would increase medical quality, reduce burnout, and mitigate the shortage of PCPs.

**Supplementary Information:**

The online version contains supplementary material available at 10.1186/s13584-024-00624-w.

## Background

Telemedicine is the use of long-distance communication to provide medical services without physical proximity between professionals and patients [1, 2]. Patients use several modalities for consulting with physicians: traditional face-to-face visits at a clinic, remote synchronous visits, such as telephone or video, and remote asynchronous visits, such as online requests. Remote visits (telemedicine) utilize a diverse range of technological possibilities, including various computer or mobile phone applications. The use of telemedicine has expanded rapidly in recent years, and many encounters that would have been conducted in person in the past now take place remotely [3]. Telemedicine has been implemented in many healthcare organizations and can potentially increase care availability and accessibility [4]. In Israel, during the COVID-19 pandemic, there was a decrease in face-to-face visits and an increase in remote visits for medical care. Many studies have shown how telemedicine can affect healthcare utilization. A cross-sectional study conducted in the United States reported that in contrast to office-based care, telemedicine was more commonly used for chronic diseases and delivery of psychiatric and behavioral treatments than for preventive care [5].

### The Israeli health system

Israel has a government-financed national healthcare system. Four health maintenance organizations (HMOs) deliver healthcare to all Israeli citizens and permanent residents who choose their HMO and may move freely from one to another. All the HMOs in Israel use electronic medical records (EMRs), and all physicians who work with an HMO have access to patient files and can review and update them.

The primary care providers are family medicine or internal medicine specialists, pediatricians, or generalists. Primary care physicians (PCPs) serve as their patients’ care coordinators. Patients choose their PCPs and are encouraged by the HMOs to use the services of their personal PCPs but may decide to be treated by other PCPs if they wish to do so. There is no co-payment for primary care visits, and the service is widely available, with most visits taking place within a few days of appointment scheduling. It is important to note that PCPs receive the same reimbursement for all visit modalities.

A patient may schedule an in-person appointment, a telephone meeting, or a video appointment with their physician. All HMOs operate digital systems that allow patients to send online requests to their PCPs. Patients may submit requests for prescriptions, referrals for laboratory tests, or sick leave certificates directly to their PCPs through these systems. In addition to these official systems, there are unofficial channels for contacting physicians, including e-mails, WhatsApp text messages, phone calls to the clinic’s administrative personnel, and other informal communication methods. The physicians may choose whether they are available to their patients via phone, video, and digital systems. The patients choose how to contact the physician with some limitations; for example, some HMOs limit the number of online requests that a patient may send or limit the availability of remote consultation to patients who had an in-person appointment within the previous 12 months, while in other cases choosing frontal modalities might often delay visit scheduling.

In Israel, there has been a significant increase in the use of telemedicine [6]. However, little is known about providers’ attitudes to the changes in their daily practices brought about by this increase. This study aimed to assess PCPs’ attitudes to the different modalities of patient care, quantify the workloads associated with the different modalities, and evaluate the appropriateness of patients’ visit modality selection.

## Methods

### Setting

This cross-sectional descriptive study was conducted with the collaboration of PCPs of all four HMOs in Israel. PCPs answered a preliminary questionnaire and documented one full day at their clinics. To obtain a convenience sample that covered the diversity of Israeli family medicine specialists and their patients, we approached physicians who worked in different parts of the country with diverse populations and were employed by the HMOs or self-employed.

### Study design

Tel-Aviv General Practice Research Network (TLV-GPRN), which gathers research-oriented family physicians, conceived and designed this study.

#### Sampling

We recruited family medicine specialists via convenience sampling.

#### Stage 1: providing instructions to the physicians

We held several teleconference meetings during which the physicians received information regarding the study process and detailed instructions for documenting a day in their working lives. Each physician attended one meeting. Those who could not participate in any meeting took part in a one-on-one instruction session with a researcher.

#### Stage 2: preliminary questionnaire

We asked the physicians to complete an online questionnaire regarding their personal details, clinic characteristics, and workload and answer 16 questions concerning their attitudes toward different modalities of medical visits.

#### Stage 3: documenting a working day at the clinic

We asked the physicians to document a full working day by summarizing all the medical visits that took place that day. We defined a medical visit as a clinical or administrative task carried out by the physician and documented in the patient’s EMR. The participating physicians used a uniform Microsoft Excel^®^ spreadsheet provided by the research coordinators for this task.

#### Day selection

We asked the physicians to select a day in July or August 2022. The chosen day was a regular working day with at least three clinical hours. To reduce recall bias, we asked the physicians to answer three visit evaluation questions immediately at the end of each visit. They were allowed to complete the patients’ sociodemographic and medical background and the visit’s characteristics later. This evaluation consisted of three parts: an answer to the question of whether the chosen visit modality was appropriate for the patient’s complaint, the perceived medical quality of the visit (on a scale of 1 to 6), and the physician’s satisfaction level after the visit (on a scale of 1 to 6). An English translation of the questionnaire is provided in the supplementary material.

Documenting a whole day was time-consuming; therefore, we advised the physicians not to choose a very long day, a day immediately following their return from a vacation, or the first working day of the week, all of which tend to be busier than other days.

### Statistical analysis

The primary outcomes were the perceived medical quality of the visits and the physicians’ feelings at the end of the visits. Both were categorized as positive (values 4–6) or negative [1–3]. We evaluated patients’ characteristics, the visit modality, and the appropriateness of the visit modality (as reported by the physician) and physicians’ and clinics’ characteristics (as reported in the Preliminary questionnaire).

We described categorical variables as frequencies and percentages and continuous non-normally distributed variables as medians and interquartile ranges (IQRs).

We compared proportions in the questionnaire answers using chi-square tests.

Since the participating physicians clustered the data regarding the visits, we used mixed effects logistic regressions to model the primary outcomes. Univariate analysis was performed with a single fixed effect.

We clustered *visit modality* into four categories: “face-to-face encounters” for in-person visits only, “remote synchronous visits” for telephone or video visits; “remote asynchronous visits” for online requests and other requests sent to the physician via various channels and handled asynchronously, and “others” that were not included in the analyses. For the variable “main issue addressed” we defined three categories: medical tasks, administrative tasks, and prescription renewal. *Administrative tasks* included sick leave, filling out various certificates, converting prescriptions and blood work asked by hospital or private physicians, etc. *Medical tasks* included acute or chronic issues and preventive medicine. *Prescription renewal* was chosen when this was the only task in the visit. Experience as a physician was clustered into four categories, balanced by their sizes: 1–7 years, 8–14 years, 15–24 years, and 25 years or older.

We included in the multivariate analysis variables associated with the outcome in univariate analyses and reported the best-fitted models in this manuscript.

All the statistical tests were two-sided, and *p* < 0.05 was considered statistically significant. Statistical analysis was performed with R version 4.1.0 (R Foundation for Statistical Computing). The study was approved by the institutional review boards of all four HMOs in Israel.

## Results

### The participants

**Fig. 1:**
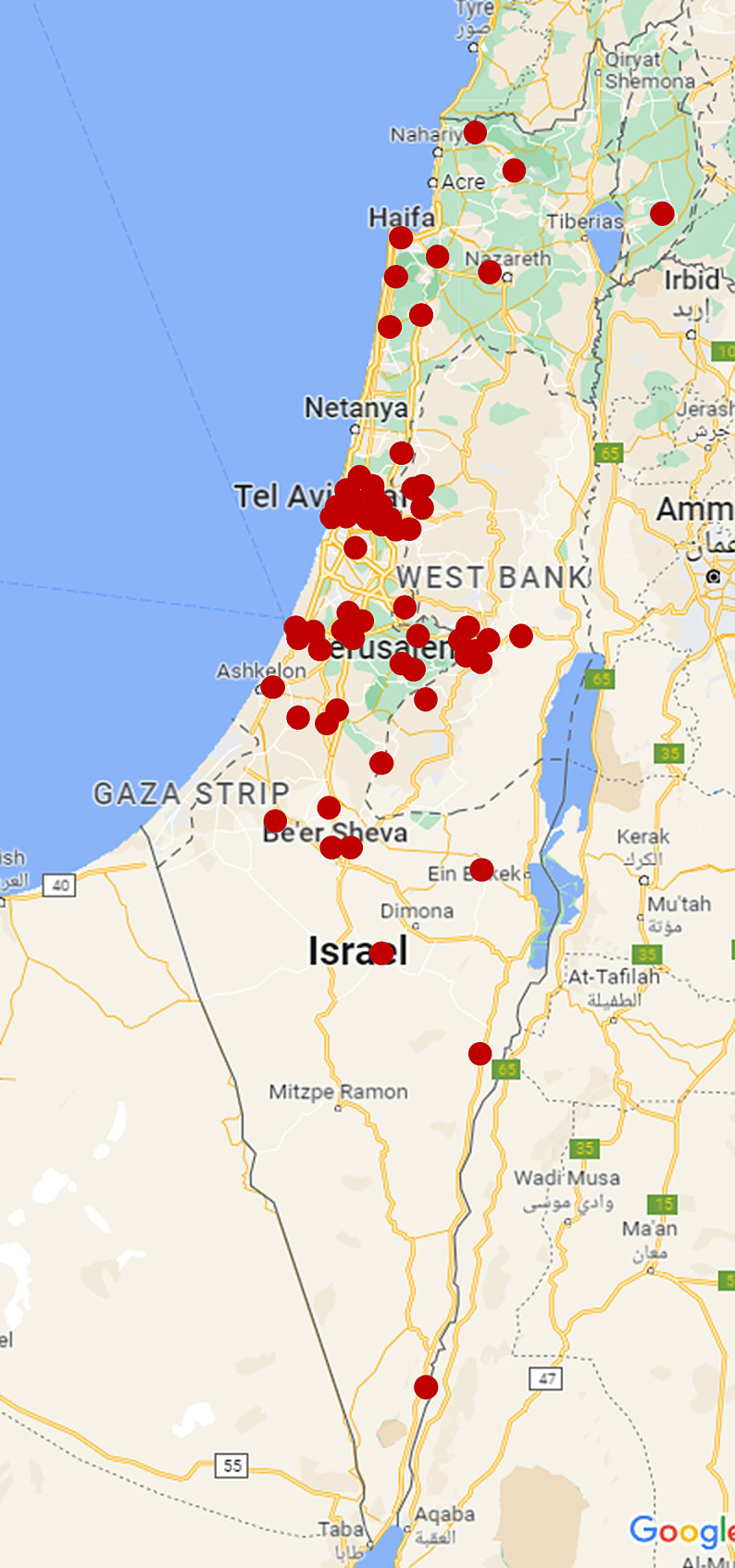
The geographical distribution of the PCPs who took part in the study

We invited 73 PCPs to participate in this study. Sixty physicians agreed to participate and documented a day in their working lives (82% response rate), and 58 answered the preliminary questionnaire. The characteristics of the participants and their clinics are detailed in Table 1, and the geographical distribution of the PCPs is presented in Fig. 1.

**Table 1:** Participating physicians and their clinics’ characteristics as reported in the questionnaire and their association with the primary outcomes (*n* = 58 responses)

Physician/clinic characteristics	*N* (%)	high-perceived medical quality^1^		positive feeling at the end of the visit^1^	
		OR	95% CI	OR	95% CI
Male (vs. female)	24 (41%)	0.96	0.52–1.77	1.19	0.65–2.17
Self-employed (vs. Salary paid)	16 (28%)	0.54	0.28–1.03	0.62	0.32–1.19
Experience as a physician (since graduation):					
1–7 years	13 (23%)	reference			
8–14 years (vs. 1–7)	16 (28%)	0.55	0.24–1.26	0.55	0.24–1.24
15–24 years (vs. 1–7)	15 (26%)	0.49	0.21–1.13	**0.40**	**0.18–0.93**
25 years and more (vs. 1–7)	13 (23%)	0.87	0.36–2.12	0.84	0.35–2.02
Clinic locale:					
Urban (vs. mixed)	40 (69%)	1.22	0.45–3.29	1.20	0.45–3.21
Rural (vs. mixed)	12 (21%)	1.48	0.47–4.63	1.52	0.49–4.74
Mixed	6 (10%)	reference			
Clinic personnel:					
Secretary	56 (97%)	0.82	0.16–4.17	1.05	0.21–5.26
Physician Assistant	3 (7%)	0.57	0.15–2.11	0.48	0.13–1.79
Nurse	51 (89%)	1.59	0.60–4.21	1.80	0.96–3.38
Resident	26 (50%)	0.65	0.35–1.22	0.78	0.41–1.44
No. of primary care physicians in the clinic (including the responding physician):					
1 physician	9 (16%)	reference			
2–3 physicians (vs. 1)	28 (49%)	1.18	0.50–2.78	1.25	0.53–2.94
4 or more physicians (vs. 1)	20 (35%)	0.68	0.28–1.68	0.86	0.35–2.13
Clinic ethnicity:					
Arabs	2 (3%)	0.68	0.10–4.55	0.44	0.07–2.85
Orthodox Jews	3 (5%)	0.52	0.10–2.62	0.48	0.10–2.41
Non-orthodox Jews	48 (83%)	0.83	0.28–2.40	0.67	0.23–1.91
Jews	5 (9%)	reference			
Socio-economic status of the clinic’s patients:					
Low (vs. medium)	7 (12%)	1.16	0.47–2.88	1.38	0.56–3.44
Medium	32 (56%)	reference			
High (vs. medium)	18 (32%)	**0.45**	**0.24–0.85**	**0.50**	**0.27–0.94**
Physicians’ practice size					
Less than 1000 patients (vs. 1000–2000)	28 (49%)	1.61	0.89–2.90	1.60	0.91–2.80
1000–2000 patients	24 (42%)	reference			
More than 2000 patients (vs. 1000–2000)	5 (9%)	**3.02**	**1.06–8.58**	**2.97**	**1.11–7.99**
Physicians’ weekly face-to-face hours:					
Less than 15 h	11 (19%)	reference			
15–20 h (vs. Less than 15 h)	11 (19%)	0.56	0.21–1.49	0.98	0.36–2.64
More than 20 h (vs. less than 15 h)	35 (61%)	0.98	0.44–2.17	1.04	0.46–2.33
Physicians’ additional roles/occupations:					
Working in more than one clinic	20 (36%)	1.09	0.58–2.06	1.29	0.68–2.42
Clinic management	17 (30%)	0.97	0.50–1.88	0.82	0.43–1.57
Other management	22 (38%)	0.66	0.36–1.22	0.68	0.37–1.25
Participation in research	28 (49%)	0.96	0.53–1.76	1.24	0.68–2.26
Teaching	51 (88%)	0.64	0.25–1.61	0.54	0.21–1.39

1. The ORs were calculated by mixed effects logistic regressions with a single fixed effect.

### Participants’ attitudes towards different modalities of medical visit, as revealed by the questionnaire

96% of the physicians who responded to the question agreed with the statement “I like to perform face-to-face consultations,” while 40% replied that they “like[d] to perform telephone consultation,” 28% answered that they “like[d] to perform video consultations.” Only 25% indicated they “like[d] to fulfill online requests” (*p* < 0.001). When asked about burnout, 81% of the physicians who responded to the question agreed with the statement “Online requests increase my burnout,” while 49%, 46%, and 44% agreed with similar statements regarding phone, video, and face-to-face consultations, respectively (*p* < 0.001). 86% of the physicians who responded to the question agreed with the statement “telephone consultations are of inferior quality compared to face-to-face consultations,” and 80% agreed with a similar statement regarding video consultations (Table 2). There was no association between physicians’ gender, years of experience, employment status, clinic SES, the number of physicians in the clinic, or the number of frontal hours that the physician worked at the clinic with any of the statements. A Higher proportion of physicians with practice smaller than 1000 patients agreed with the statements that remote synchronous and asynchronous visits decrease their workload (*p* = 0.02, *p* = 0.03, respectively).

**Table 2:** Participating physicians’ attitudes toward different modalities of medical visits

Statement (by order of agreement)	Agreed *n* (%)
I like to conduct face-to-face consultations. (57 responses)	55 (96.5)
Telephone consultations are inferior to face-to-face consultations. (58 responses)	50 (86.2)
Online requests are beneficial to the patient. (57 responses)	48 (84.2)
Online requests increase my burnout. (57 responses)	46 (80.7)
Video consultations are inferior to face-to-face consultations. (56 responses)	45 (80.4)
A skilled physician can also conduct quality video consultations. (57 responses)	36 (63.2)
A skilled physician can also conduct quality telephone consultations. (58 responses)	36 (62.1)
Online requests decrease my workload as a physician. (58 responses)	35 (60.3)
Online requests are efficient from the physician’s perspective. (58 responses)	34 (58.6)
Telephone consultations increase my burnout. (57 responses)	28 (49.1)
Video consultations increase my burnout. (46 responses)	22 (45.8)
Face-to-face consultations increase my burnout. (57 responses)	25 (43.9)
Telephone/video consultations decrease my workload as a physician. (58 responses)	24 (41.4)
I like to conduct telephone consultations. (57 responses)	23 (40.4)
I like to conduct video consultations. (50 responses)	14 (28.0)
I like to fulfill online requests. (57 responses)	14 (24.6)

### The documented day

**Fig. 2:**
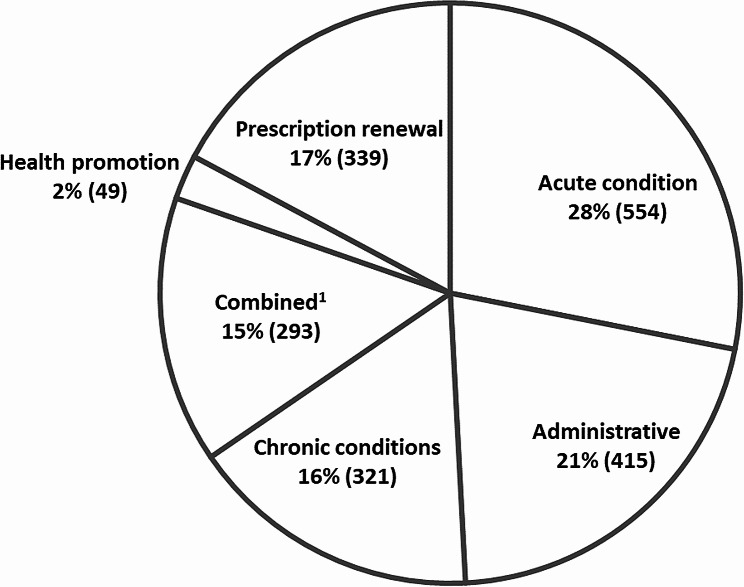
The main issues addressed during 1,971 visits (missing data for 54 visits)

Sixty physicians documented 2,025 visits. The median number of visits that a physician performed during the documented day was 31 (interquartile range [IQR] 25–40); the visits’ distribution among the different modalities was as follows: 784 (39%) face-to-face visits, 730 (36%) online requests, 373 (18%) telephone visits, 13 (< 1%) video visits, 66 (3%) physician-initiated visits, and 58 (3%) other types of visits. Physicians reported that the patient chose an inappropriate modality for 486 (24%) visits. The main issues addressed during the visits are presented in Fig. 2.

**Fig. 3:**
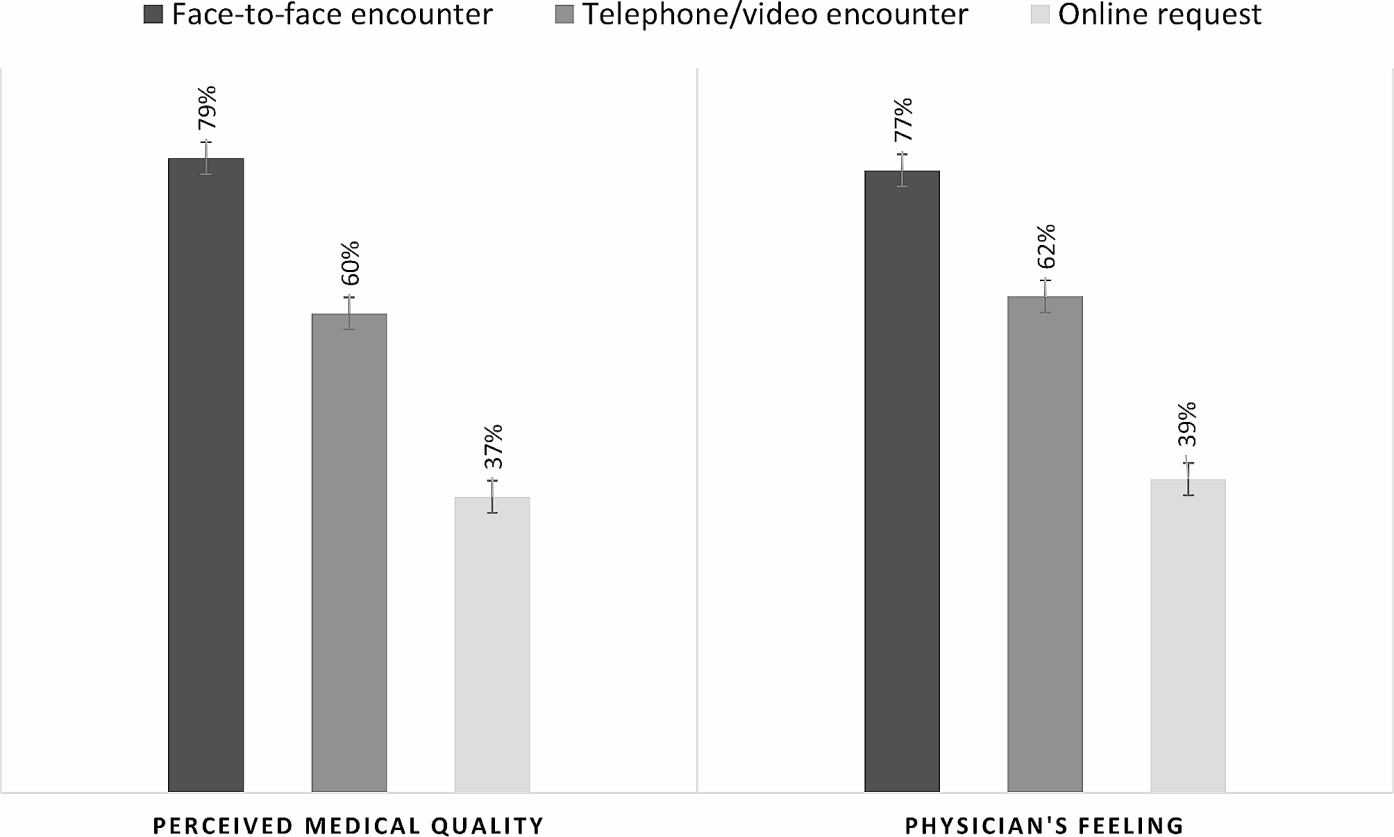
Proportion of visits with high-perceived medical quality (left) and positive feelings at the end of the visit (right)

The physicians perceived 79% of the face-to-face visits as high medical quality compared to 60% and only 37% of remote synchronous visits (telephone/video) and remote asynchronous visits (online requests), respectively. Similarly, the proportions of visits that ended with a positive feeling were 77%, 62%, and 39%, respectively (Fig. 3).

Of the physicians’ and clinics’ characteristics reported in the preliminary questionnaire, only a few were associated in univariate analyses with high-perceived medical quality or positive feelings at the end of the visit. Physicians who worked in a clinic with high SES patients had lower odds of positive feelings or high medical quality (OR = 0.50, 95% CI 0.27–0.94 and OR = 0.45, 95% CI 0.24–0.85, respectively). Physicians with practice size larger than 2000 patients had higher odds for positive feelings or high medical quality (OR = 2.97, 95% CI 1.11–7.99 and OR = 3.02, 95% CI 1.06–8.58 respectively) (Table 1).

### Multivariate analysis

Mixed effects logistic regressions were used to model the visits’ evaluation (Table 3). Perceived medical quality and the feeling at the end of the visit were both associated with the visit modality, the main issues addressed, the physician’s perception of the visit’s modality appropriateness, and the visit order during the day. We found no association between the perceived medical quality or the feeling at the end of the visit and patients, physicians, or clinic characteristics.

### Perceived medical quality

When the physician perceived the visit modality as improper, the odds for perceived high medical quality were lower (aOR-0.13, 95% CI 0.09–0.18). The timing of the visit was also associated with high medical quality; the odds increased as the visit took place later during the documented day (OR-1.01, 95% CI 1.003–1.03).

We found interactions between the visit modality and the main issue addressed. Face-to-face visits focused on administrative tasks and prescription renewal were perceived as of lower medical quality than face-to-face visits focused on medical tasks (OR-0.07, 95% CI 0.04–0.15, and OR-0.03, 95% CI 0.01–0.08, respectively). Comparing the different visit modalities for medical tasks, physicians perceived remote synchronous or asynchronous visits as lower medical quality than face-to-face visits (OR-0.39, 95% CI 0.25–0.59, and OR-0.14, 95% CI 0.09–0.23). This is also true for administrative tasks: physicians perceived remote asynchronous visits as having lower medical quality than face-to-face visits (OR-0.31, 95% CI 0.14–0.65). On the contrary, physicians perceived prescription renewals done in remote synchronous visits as having higher medical quality than those done in face-to-face visits (OR-3.91, 95% CI 1.02–14.97).

### Physicians’ feelings at the end of the visit

There was a strong correlation between the perception of medical quality and the physicians’ feelings at the end of the visits (both reported on a scale of 1 to 6), Spearman’s *r* = 0.82 (*p* < 0.001). The multivariate analysis of physicians’ feelings at the end of the visit is presented in the supplementary material (Table 1s).

**Table 3:** Mixed effects logistic regressions for perceived medical quality

	Odds ratio	95% confidence interval
**Visit modality**		
face-to-face visit		
Administrative tasks vs. medical tasks	0.07	0.04–0.15
Prescription renewal vs. medical tasks	0.03	0.01–0.08
Remote synchronous visit (telephone/video)		
Administrative tasks vs. medical tasks	0.1	0.05–0.21
Prescription renewal vs. medical tasks	0.32	0.11–0.94
Remote asynchronous visit (online requests)		
Administrative tasks vs. medical tasks	0.16	0.09–0.28
Prescription renewal vs. medical tasks	0.24	0.14–0.42
**Main issue addressed**		
Medical tasks		
Remote synchronous vs. face-to-face	0.39	0.25–0.59
Remote asynchronous vs. face-to-face	0.14	0.09–0.23
Administrative tasks		
Remote synchronous vs. face-to-face	0.51	0.21–1.28
Remote asynchronous vs. face-to-face	0.31	0.14–0.65
Prescription renewal		
Remote synchronous vs. face-to-face	3.91	1.02–14.97
Remote asynchronous vs. face-to-face	1.08	0.41–2.82
**An inappropriate visit modality (vs. an appropriate modality)**	0.13	0.09–0.18
**Visit order during the day**	1.01	1.003–1.03

## Discussion

Our findings show that remote asynchronous visits (online requests) have become a prominent part of the daily work of PCPs in Israel, accounting for 36% of all PCP visits in this study. Classic face-to-face visits comprised only 39% of PCPs’ visits, and remote synchronous visits (telephone/video) accounted for approximately 20%. A substantial portion of the physicians’ daily visits was dedicated to administrative work. The participating physicians evaluated remote asynchronous visits as of low medical quality and were unsatisfied with them. The COVID-19 pandemic has changed the character of patient–physician encounters and dramatically increased telemedicine use. Most PCPs in Israel conducted face-to-face and remote visits during the pandemic, and approximately 40% had no previous telemedicine experience [7]. Remote visits in primary care also increased dramatically in Canada [8], the United States [9, 10], Mexico [11], and many other countries. Many PCPs experienced telemedicine for the first time during the pandemic [12]. Although we conducted this study months after all the COVID-19 restrictions were lifted and the incidence of the disease dropped, only 39% of patient-physician visits in this study occurred in person.

Administrative tasks took up a significant portion of PCPs’ time and comprised 21% of all encounters in our study. Substantial administrative work by PCPs has been reported previously. A study in the US found that family physicians devoted 17% of their total work to administrative tasks [13]. In another study, physicians from diverse specialties spent 7.7% of their time on administrative work [14].

Our study demonstrated that physicians perceived remote visits as a more significant contributor to their burnout than face-to-face visits, with remote asynchronous visits being the most important, while only those with a small practice size think that remote visits reduce their workload. In a large survey among physicians, family physicians ranked fourth out of 29 specialties on burnout rate, with 51% of PCPs reporting feeling burned out in 2021 [15]. Many factors have been found to be related to physician burnout. PCPs working in solo practices were less likely to report burnout than those in larger practices. Efficient teamwork has been found to be associated with a lower burnout rate [16]. PCPs’ burnout was not found to differ between rural and urban practices [17]. Burnout rates were found to be higher among female PCPs than among their male counterparts [18]. We did not find associations between physicians’ or clinics’ characteristics and the perception of burnout.

A significant gap exists between physicians’ and patients’ preferences for visit modality. While physicians preferred face-to-face visits and rated their quality much higher than remote synchronous or asynchronous visits, patients frequently chose remote communication.

The multivariate analysis revealed the complexity, specifically in the interactions between variables. When evaluating how physicians perceived the medical quality of their visits, several factors emerged as significant: the visit modality, the main issue addressed in the visit, the appropriateness of the visit modality, and the timing of the visit.

The documented day analysis supported the finding of the preliminary questionnaire that physicians prefer face-to-face visits to telemedicine. This preference was relevant for visits for medical tasks and, surprisingly, administrative tasks. Another important finding was physicians’ preference for medical tasks (in all the modalities) rather than administrative tasks or prescription renewals. As the Israeli HMOs compensate self-employed physicians for visits regardless of their modality, we anticipated that self-employed physicians would have a weaker preference (if any) for face-to-face visits or medical tasks since they consume more time than remote visits or administrative tasks. However, we found no association between employment status and the outcomes.

When the physician felt that the modality chosen by the patient was improper, the perceived medical quality and the feeling at the end of the visit were low.

### Strengths and limitations

The study has some limitations. Firstly, its sample did not represent the PCPs in Israel, as it included only family medicine specialists, who comprised in 2018 32% of the PCPs in Israel [19]. Men’s proportion in this sample was similar to their proportion among family medicine specialists in 2018; we were unable to compare other characteristics. Secondly, the physicians subjectively evaluated their visits, while an objective evaluation could not be obtained. Nevertheless, the sample included a leading group of PCPs working for the four Israeli HMOs in different areas around the country and with different employment statuses. They and their patients represent the diversity of the Israeli population. The large number of visits documented in real-time created a unique opportunity to access unbiased physicians’ perspectives on these visits and their working days. We’d asked the physicians to report all the encounters they had during the day. We’ve received no indication of partial reporting, and the reported amounts of visits were within the expected range.

### Implications

Our study points to a gap between the high volume of remote synchronous and asynchronous medicine, the perceived medical quality, and physicians’ sentiment. While most physicians enjoy personal interactions during face-to-face visits, they spend more and more time on remote visits and administrative work. This gap may explain the frustration and high burnout rates among PCPs.

Administrative tasks and prescription renewals are time-consuming and contribute to PCPs’ burnout. The Ministry of Health (MoH) and the HMOs should seek ways to decrease these tasks’ volume and facilitate their execution for physicians. HMOs should reduce the need for administrative tasks such as filling forms and conversion of prescriptions or referrals for blood work and imaging given by other physicians. These tasks are time-consuming and rarely require the PCP’s medical judgment. Another possible solution may be automated computerized systems that replace or at least facilitate the physicians’ work on selected administrative tasks. As the general shortage of physicians, especially PCPs becomes a critical challenge in Israel, load reduction would free time for the existing physicians and partially mitigate this challenge.

The visit modality is chosen by the patients; an automated computerized triage system operating at appointment scheduling would direct the patients to a visit modality optimal for their complaints. The Israeli Association of Family Physicians should develop guidelines for the optimal modality for a complaint that the HMOs would later implement.

## Conclusion

In this study, physicians preferred face-to-face visits compared to remote visits when medical or administrative tasks were the main issue of the visit. They dedicated a significant proportion of their working time to fulfilling online requests and carrying out administrative tasks. They found these visits to be of low medical quality and felt much less satisfied upon their completion. Policymakers should intervene to minimize administrative work, reduce the PCP’s workload, and direct patients to the optimal visit modality for their complaints. These steps would increase medical quality, reduce burnout, and mitigate the shortage of PCPs.

### Electronic supplementary material

Below is the link to the electronic supplementary material.

Supplementary Material 1

Supplementary Material 2

## Data Availability

The data that support the findings of this study are available upon request from the corresponding author, GZ, or LA. The data are not publicly available due to ethical restrictions.

## Abbreviations

PCPs: Primary care physicians
OR: Odds ratio
HMOs: Health maintenance organizations
EMRs: Electronic medical records
IQRs: Interquartile ranges
