# Supplementary material for: “A day in the life” – telemedicine in family medicine and its relationship with practicing physicians’ satisfaction: a cross-sectional study
Source: Isr J Health Policy Res. 2024 Jul 29;13:33. doi: 10.1186/s13584-024-00624-w (PMC11287843; doi:10.1186/s13584-024-00624-w)
Supplement: Supplementary file 1 — Supplementary Material 1 [file 13584_2024_624_MOESM1_ESM.docx]

**Questionnaire of physician and clinic characteristics**

1. Name (the questionnaire was not anonymous as it was later linked with visits information)

2. Gender

3. Seniority as a physician (years since beginning residency/specialization training)

4. Clinic locale - urban/rural/mixed

5. Employment format at the primary care clinic - salaried/ self-employed/ self-employed in a group practice

6. How many physicians work together at your clinic?

7. Does your clinic employ a secretary yes/no Nurse yes/no, Physician assistant yes/no, Resident (trainee) yes/no

8. What is the mean socio-economic level of your clinic’s patients? Low/moderate/high

9. What is the ethnic background of most of your clinics patients? Jewish/ Arab/ orthodox Jewish (if over 30% of population is in group, more than one choice is possible)

10. How many patients belong to your personal practice? (less than 1000 / 1000-2000 / over 2000)

11. How many weekly "frontal" (face to face) hours do you work in your clinic? Up to 10 / 10 – 15 / 15-20 / Over 20)

12. Do you work in clinical care in another clinic? Yes/No

13. Do you work in clinical care in another specialization? Yes/No

14. Do you work in clinical care in a hospital? Yes/No

15. In addition to your clinical work do you also work in clinic management? Yes/No

16. In addition to your clinical work do you also work in management (Not related to the clinic)? Yes/No

17. Do you participate in research? Yes/No

18. Do you participate in teaching residents (trainees) and/or medical students in Family Medicine? Yes/No

19. The following questions please rate on a scale from 1 – 6 (1 Don't agree; 6 Highly Agree)

- On line requests / digital requests lessen my workload as a physician.

- Telephone/Video visits lessen my workload as a physician.

- I think a skilled physician can also perform quality visits by video.

- I think a skilled physician can also perform quality visits by telephone.

- I think telephone visits are of poorer quality than visits in person

- I think video visits are of poorer quality than visits in person

- On line requests / digital requests are more efficient for the physician

- On line requests / digital requests are more efficient for the patient

- On line requests / digital requests wear me out

- Telephone visits wear me out

- Video visits wear me out

- Frontal (face to face) visits wear me out

- I like to perform telephone visits

- I like to perform video visits

- I like to perform frontal (face to face) visits

- I like to perform online requests / digital requests visits
