# Supplementary material for: “A day in the life” – telemedicine in family medicine and its relationship with practicing physicians’ satisfaction: a cross-sectional study
Source: Isr J Health Policy Res. 2024 Jul 29;13:33. doi: 10.1186/s13584-024-00624-w (PMC11287843; doi:10.1186/s13584-024-00624-w)
Supplement: Supplementary file 2 — Supplementary Material 2 [file 13584_2024_624_MOESM2_ESM.docx]

**Table 1s. Mixed effects logistic regressions for physicians’ feelings at the end of the encounters**

|  | **Odds ratio** | **95% confidence interval** |
| --- | --- | --- |
| **Encounter modality** | | |
| Face-to-Face encounter |  |  |
| Administrative tasks vs. medical tasks | 0.12 | 0.06-0.22 |
| Prescription renewal vs. medical tasks | 0.11 | 0.05-0.26 |
| Remote synchronous encounter (telephone/video) |  |  |
| Administrative tasks vs. medical tasks | 0.10 | 0.05-0.20 |
| Prescription renewal vs. medical tasks | 0.48 | 0.18-1.32 |
| Remote asynchronous encounter (online requests) |  |  |
| Administrative tasks vs. medical tasks | 0.23 | 0.14-0.40 |
| Prescription renewal vs. medical tasks | 0.30 | 0.18-0.51 |
|  | | |
| **Main issue addressed** |  |  |
| Medical tasks |  |  |
| Remote synchronous vs. Face-to-Face | 0.54 | 0.36-0.81 |
| Remote asynchronous vs. Face-to-Face | 0.18 | 0.11-0.29 |
| Administrative tasks |  |  |
| Remote synchronous vs. Face-to-Face | 0.45 | 0.19-1.08 |
| Remote asynchronous vs. Face-to-Face | 0.36 | 0.18-0.72 |
| Prescription renewal |  |  |
| Remote synchronous vs. Face-to-Face | 2.27 | 0.67-7.69 |
| Remote asynchronous vs. Face-to-Face | 0.48 | 0.20-1.09 |
|  | | |
| **An inappropriate encounter modality (vs. an appropriate modality)** | 0.16 | 0.12-0.23 |
|  | | |
| **Visit order during the day** | 1.02 | 1.01-1.03 |
